# RLEAAI: improving antibody–antigen interaction prediction using protein language model and sequence order information

**DOI:** 10.1093/bib/bbaf238

**Published:** 2025-06-04

**Authors:** Jun Hu, Yu Zhou, Wen-Yi Zhang, Xiao-Gen Zhou

**Affiliations:** College of Information Engineering, Zhejiang University of Technology, 288 Liuhe Road, Liuxia Street, Xihu District, Hangzhou 310023, China; Center for AI and Computational Biology, Suzhou Institute of Systems Medicine, 100 Chongwen Road, Suzhou 215123, China; College of Information Engineering, Zhejiang University of Technology, 288 Liuhe Road, Liuxia Street, Xihu District, Hangzhou 310023, China; Westlake AI Therapeutics Lab, Westlake Laboratory of Life Sciences and Biomedicine, 18 Shilongshan Road, Hangzhou, Zhejiang 310024, China; College of Information Engineering, Zhejiang University of Technology, 288 Liuhe Road, Liuxia Street, Xihu District, Hangzhou 310023, China

**Keywords:** antibody, antigen, interactions, protein language model

## Abstract

Antibody–antigen interactions (AAIs) are a pervasive phenomenon in the natural and are instrumental in the design of antibody-based drugs. Despite the emergence of various deep learning-based methods aimed at enhancing the accuracy of AAIs predictions, most of these approaches overlook the significance of sequence order information. In this study, we propose a new deep learning-based method RLEAAI, to improve the prediction performance of AAIs. In RLEAAI, a sequence order extraction strategy, called Composition of K-Spaced Amino Acid Pairs, is employed to generate feature representation from the feature embedding outputted by a pre-trained protein language model. In order to fully dig out the discrimination information from features, three neural network modules, i.e. convolutional neural network, bidirectional long short-term memory network and recurrent criss-cross attention mechanism, are integrated. Benchmarked results on two independent test sets demonstrate that RLEAAI is capable of achieving an average accuracy of 0.7787 and an average Matthews’s correlation coefficient (MCC) value of 0.5552, representing a 5.2% and 15.8% improvement over the start-of-the-art method DeepAAI. Furthermore, the complementary determining regions-sensitivity value calculated on MCC of RLEAAI is 216.4% higher than that of the state-of-the-art method DeepAAI. The standalone package of RLEAAI is freely available at https://github.com/zhouyu9931/RLEAAI.git.

**Key Points**

- RLEAAI employs a sequence order extraction strategy of Composition of K-Spaced Amino Acid Pairs to generate feature representation from the feature embedding outputted by ESM2 language model.
- Experimental results on two datasets show that RLEAAI is able to predict antibody–antigen interactions more accurately than other state-of-the-art methods.
- When different complementary determining regions (CDRs) are masked, RLEAAI demonstrates greater sensitivity to CDRs compared to other existing or state-of-the-art methods, aligning with the mechanism of AAIs.

## Introduction

Antibodies are proteins generated in response to an invading pathogen and are crucial components of the immune system [1, 2]. It is estimated that the human body can produce ~10^20^ different antibodies to combat viral infections [3], highlighting the significant role of antibody–antigen interactions (AAIs) in the immune process. The binding specificity between antibodies and antigens is the central mechanism by which the immune system recognizes and is vital for disease treatment and the development of antibody-based drugs [4]. Recent studies have provided deeper insights into the structural basis of AAIs, highlighting the crucial role of complementarity between antigenic epitopes and antibody paratopes in ensuring high specificity [5, 6]. Researchers have continually explored antibody preparation methods and have made efforts to apply these methods in clinical therapy [7–9]. Given the crucial role of antibody–antigen specificity in the context of immunoassays [10]. Radioimmunoassays and enzyme-linked immunosorbent assays (ELISAs) have been extensively employed to assess the affinity and specificity of AAIs [11–13]. The key to improving antibody drug design lies in the accurate identification of AAIs, which can be validated through traditional wet lab experiments, including phage display [14], ELISA [11], and pseudovirus detection [15], though this process is both expensive and time-consuming [16, 17]. Advanced methodologies such as high-throughput epitope mapping and engineered antibody libraries have addressed limitations in traditional immunoassays [18, 19]. Large-scale meta-analyses highlight the clinical relevance of AAIs in vaccine development and autoimmune disease diagnostics [20]. In recent years, computational methods have emerged as a promising approach for predicting AAIs, with the potential to facilitate antibody optimization and antibody drug design. These methods rely on the fundamental principle of deriving insights from known AAIs to predict unknown AAIs [21, 22]. By analyzing existing data, computational approaches aim to enhance the accuracy and efficiency of predicting AAIs, offering valuable support for advancing drug development and therapeutic strategies [23].

Many deep learning-based methods have been developed to identify AAIs [24], such as AbAgIntPre [25], DeepAAI [2, 6], and S3AI [27]. Concretely, AbAgIntPre utilizes an encoding scheme based on the amino acid composition of antigens and antibodies to construct a Siamese-like convolutional neural network (CNN) for rapid identification of AAIs that solely depend on amino acid sequences. However, the depth of the neural network used by AbAgIntPre is not enough, rendering it ineffective at extracting complex information from the sequences. DeepAAI improves upon this by constructing two adaptive relational graphs that connect antibody and antigen sequences, applying Laplacian smoothing to learn the representations of antibodies. In the relational graph, the graph convolutional network (GCN) [28] is used to process the information embedded in the nodes (representations of antibodies and antigens) and edges (quantitative relationships between antibody sequences and between antigen sequences). S3AI takes a different approach by automatically incorporating implicit structural information and chemical constraints while relying solely on sequence inputs to enhance the prediction performance of AAIs. Recently, AbAgIPA [29] effectively captures intricate structural features of AAIs using backbone-aware representations and invariant point attemtion (IPA), but its reliance on accurate structure prediction may limit its applicability in certain scenarios. Although the above methods have achieved some success, they do not pay enough attention to the order information of the sequence, which limits the performance of AAI’s prediction.

Recent advances in the large-scale protein language models, such as Prot5_XL [30] and ESM2 [31], have made significant contributions in the research fields of protein structure prediction, protein–protein interactions prediction [32], and AAIs prediction [25]. The well-known pretrained protein language model, i.e. ESM2 [31] have demonstrated the potential of leveraging extensive protein sequence data to enhance capabilities of capturing protein semantic information. ESM2 is trained on UniRef50 [33] and stands out for its widespread application in the field of protein interactions. Fully mining the discriminative information from the feature embedding outputted by ESM2 is one of the feasible solutions to improve the AAIs’ prediction performance.

In this study, we employ ESM2 to extract the feature representations from antibody and antigen sequences. To extract more discriminative information from feature representations, we incorporate the recurrent criss-cross attention mechanism (RCCA) [34] and the Composition of K-Spaced Amino Acid Pairs (CKSAAP) [35] method to capture global contextual information effectively by repeatedly computing attention weights along the rows and columns. This mechanism not only significantly enlarges the receptive field of the model but also selectively focuses on important features relevant to the target. Except for RCCA, the modules of CNN and bidirectional long short-term memory network (BiLSTM) are also employed to dig out discriminative information from the ESM2-based features. Experimental results on the independent testing datasets concerning two different virus types, i.e. HIV and Severe Acute Respiratory Syndrome Coronavirus 2 (SARS-CoV-2), demonstrate that RLEAAI achieves an average prediction accuracy of 0.7787 and an average Matthews’s correlation coefficient (MCC) of 0.5552, outperforming other state-of-the-art AAI prediction methods. In addition, RLEAAI is more sensitive to the changes of CDR regions against to other existing AAIs prediction methods.

## Materials and methods

### Benchmark datasets

Three datasets are employed in this study to evaluate the performance of the proposed RLEAAI. The first is HIV dataset collected in DeepAAI [26], which is procured from the Compile, Analyze and Tally Nab Panels [36] at the Los Alamos HIV Database [37]. There are two subsets in HIV dataset divided in DeepAAI, i.e. training (HIVtr) and testing (HIVtst) datasets, which contain 24 843 and 4551 samples, respectively. Here, one sample means one pair of antigen and antibody sequences with the corresponding label. The positive/negative label means the corresponding antigen and antibody interacted/non-interacted with each other. In this study, to more rigorously assess the generalization ability of the proposed RLEAAI, we extra used two sequence identity cutoffs of 0.9 and 0.85 to remove the redundant samples on the test subset against the training subset, resulting to produce two new testing sets, i.e. HIVtst90 and HIVtst85. Concretely, two redundant samples should contain redundant antibody sequences, redundant antigen sequences, and same label. It is noted that there are a few independent testing samples when the sequence identity cutoff is set to 0.8. The detailed distributions of the dataset are shown in Table 1.

**Table 1:** Distribution of the HIV datasets in this study.

Datasets	${\mathrm{N}}_{\mathrm{pos}}$	${\mathrm{N}}_{\mathrm{neg}}$	${\mathrm{N}}_{\mathrm{total}}$
HIVtr	12 615	12 228	24 843
HIVtst	1897	2564	4551
HIVtst90	1522	2075	3597
HIVtst85	1240	1595	2835

${\mathrm{N}}_{\mathrm{pos}}$ means the number of positives samples. ${\mathrm{N}}_{\mathrm{neg}}$ means the number of negative samples. ${\mathrm{N}}_{\mathrm{total}}$ means the number of total samples.

The second dataset is collected in this study, called SARS-CoV-2, for the performance of the proposed RLEAAI on different types of viruses. Specifically, for each sample, antibody sequence and the corresponding virus sequence are collected from the CovAbDab database [38]. All virus sequences are sourced from the National Center for Biotechnology Information (NCBI) database [39], which includes variants of SARS-CoV-2, i.e. Alpha, Beta, Delta, Gamma, and Omicron. The dataset is divided into a training set (CoVtr) consisting of 6150 samples and a test set (CoVtst) containing 754 samples. The distribution of the datasets is illustrated in Table 2.

**Table 2:** Distribution of the SARS-CoV-2 data in this study.

Datasets	${\mathrm{N}}_{\mathrm{pos}}$	${\mathrm{N}}_{\mathrm{neg}}$	${\mathrm{N}}_{\mathrm{total}}$
CoVtr	2979	3171	6150
CoVtst	397	357	754

${\mathrm{N}}_{\mathrm{pos}}$ means the number of positives samples. ${\mathrm{N}}_{\mathrm{neg}}$ means the number of negative samples. ${\mathrm{N}}_{\mathrm{total}}$ means the number of total samples.

The third dataset is a five-fold cross-validation dataset constructed by AbAgIPA [29] based on the SAbDab database. Following the methodology outlined in AbAgIntPre, AbAgIPA utilizes CD-HIT for redundancy removal, where antigens with sequence identity exceeding 90% are considered identical. AAI pairs associated with these antigens are grouped into the same subset, resulting in 3800 positive samples. For each antigen represented in the positive samples, negative samples are generated by pairing it with antibodies sampled from different subsets, yielding a total of 3800 negative samples.

### Pipeline of RLEAAI

**Figure 1:**
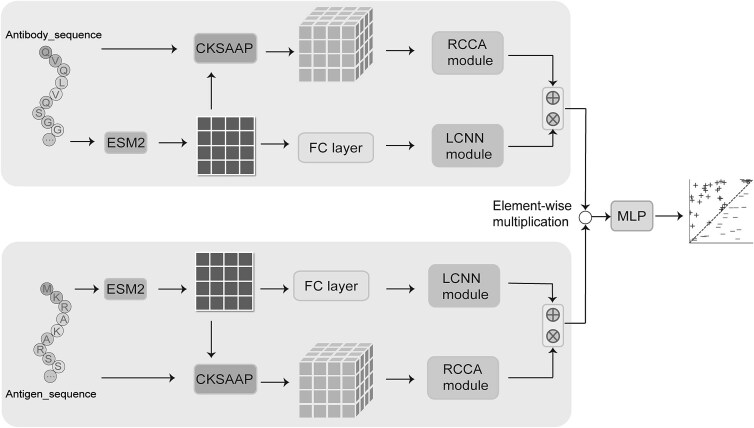
The overall architecture of RLEAAI.

In this study, we introduce a deep learning framework, called RLEAAI, to improve the performance of AAI’s prediction. As illustrated in Fig. 1, the query sequence pair of antibody and antigen is first processed through the pre-trained protein language model ESM2 to generate two feature representations corresponding to antibody and antigen, respectively. The query antibody and antigen sequences are fed into the pre-trained ESM2 model, which generates an embedding corresponding to each sequence. The embedding encapsulates the essential features of the sequences, allowing the model to capture complex relationships within and between the antibody and antigen sequences for further processing. For each amino acid position, a high-dimensional vector is generated, representing the features at that specific location. The two representations are separately fed into two different neural network modules: the first module is composed of one sequence order extraction strategy (called CKSAAP) sub-module and one RCCA module and the second module consists of one fully connected liner layer and the local convolutional neural network (LCNN) sub-module, which serially combines CNN and BiLSTM layers. The outputs of both modules are fused to form a more comprehensive and discriminative representation of the sequence features. Finally, the combined information is fed into the multilayer perceptron (MLP) layer to make task-specific predictions. This integration of different feature processes enhances the ability to detect complex patterns in AAIs.

### Convolutional neural network

The CNN [40] architecture is employed in this study to extract the local relationship information embedded in antigen and antibody sequences. In this context, let $X=\left[{x}_1,{x}_2,{x}_3,\dots \dots, {x}_l\right]$ is used to denote the input corresponding to the output of the previous neural network layer. The convolutional layer employs the convolutional kernel as a sliding window to generate a sequence of latent vectors $H=\left[{h}_1,\kern0.5em {h}_2,\dots, {h}_{l-m+1}\right]$, where each ${h}_t$ is computed as follows:


(1) \begin{equation*} {h}_t= Conv\left({x}_{t:t+m-1}\right) \end{equation*}

where *l* is the length of the antigen/antibody sequence and *m* is the size of the convolutional kernel.

### Bidirectional long short-term memory network

Long short-term memory network (LSTM) [41] represents a specific category of recurrent neural network (RNN) [42], designed to capture long-term dependencies by maintaining an internal memory cell. Each unit is comprised of three distinct types of gates: forget gate $\left({f}_t\right)$, input gate $\left({i}_t\right)$, and output gate $\left({o}_t\right)$. The forget gate is responsible for determining which information should be discarded or retained. The input gate is utilized to update the state of the cell, while the output gate determines the value of the subsequent hidden state. LSTM updates the previous state ${h}_{t-1}$ of the sequence to the hidden state ${h}_t$, calculated as follows:


(2) \begin{equation*} {f}_t=\sigma \left({W}_f\bullet \left[{h}_{t-1},{x}_t\right]+{b}_f\right) \end{equation*}

(3) \begin{equation*} {i}_t=\sigma \left({W}_i\bullet \left[{h}_{t-1},{x}_t\right]+{b}_i\right) \end{equation*}

(4) \begin{equation*} {\overset{\sim }{C}}_t=\mathit{\tanh}\left({W}_c\bullet [{h}_{t-1},{x}_{t}]+{b}_C\right) \end{equation*}

(5) \begin{equation*} {C}_t={f}_t\ast{C}_{t-1}+{i}_t\ast{\overset{\sim }{C}}_t \end{equation*}

(6) \begin{equation*} {o}_t=\sigma \left({W}_o\bullet \left[{h}_{t-1},{x}_t\right]+{b}_o\right) \end{equation*}

(7) \begin{equation*} {h}_t={o}_t\ast \mathit{\tanh}\left({C}_t\right) \end{equation*}

where *W* denotes different weight matrices, *b* denotes different biases, ${C}_t$ represents memory of the LSTM at the current time step, ${\overset{\sim }{C}}_t$ represents new candidate values for the cell state, $\mathit{\tanh}$ is activation function constraining the values between −1 and 1 and $\sigma$ is the sigmoid function.

BiLSTM enhances the capabilities of traditional LSTM by processing sequence data in both forward and backward directions. This bidirectional approach allows the model to incorporate both past and future context, resulting in a more comprehensive representation of global features and long-range dependencies within the sequence. For each time step, the outputs from the forward LSTM layer and the backward LSTM layer are combined to form an integrated representation as ${h}_t= BiLSTM\left({x}_t\right)=\left[\overrightarrow{LSTM}\left({x}_t\right),\overleftarrow{LSTM}\left({x}_t\right)\right]$*.*

### LCNN module

**Figure 2:**
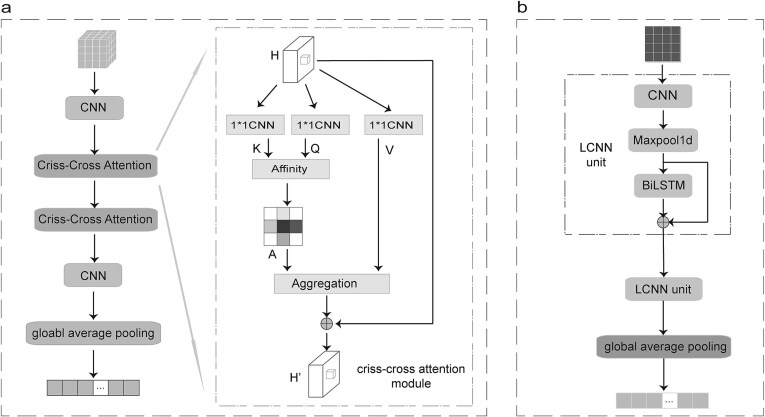
Two modules. (a) Details of RCCA module. (b) Details of LCNN module.

The LCNN module is mainly composed of *n* units with same architecture. As shown in Fig. 2b, each unit integrates three different neural network layers, i.e. CNN, max-pooling, and BiLSTM. It is noted that the residual mechanism is employed to control the learning speed of BiLSTM. The CNN and BiLSTM layers can mainly detect the local patterns and long-range dependencies within a sequence, respectively, resulting LCNN to catch more sequence discriminative information for the prediction task in this study, i.e. AAIs classification. In this study, *n* is set to 2, which is tuned on both HIV and SARS-CoV-2 data.

### RCCA module with CKSAAP

To extract more sequence order information, we combine the sequence order information extraction strategy, i.e. CKSAAP (see Supplementary Text S1), and recurrent RCCA, called RCCA, to help us dig out more discriminative information from the feature embedding generated by ESM2. The CKSAAP-generated feature is a type of feature representation method based on amino acid pairs in a protein sequence. It captures information about the relative positions and spacing of amino acids in the sequence. Specifically, the CKSAAP feature represents the frequency distribution of amino acid pairs that are *k* positions apart in the sequence, which can directly represent the sequence order information. By computing the frequency of amino acid pairs at different *k* values, a high-dimensional feature vector can be constructed. Additionally, the RCCA module incorporates an RCCA, which further focuses on the sequence order information related to residues. The CKSAAP features are generated by combining the protein sequences with the embedding generated by ESM2. The final dimension of the generated feature is $1280\times \left(k+1\right)\times 20\times 20$, where *k* represents the spacing distance in the sequence.

The RCCA mechanism improves upon traditional transformer architectures by calculating attention weights for each amino acid along both row and column directions. This dual-directional attention allows each amino acid to effectively integrate contextual information from other amino acids across the sequence. Unlike the global self-attention mechanism in transformers, which can be computationally expensive and less tailored for specific structural dependencies, the RCCA focuses on more targeted feature interactions. By weighting and summing features across distinct row and column directions, it emphasizes the most relevant features for each amino acid, offering a more specialized and efficient approach for capturing interdependencies in amino acid sequences. As shown in Fig. 2a, the criss-cross attention firstly applies three convolutional layers with $1\times 1$ filters to the input features *H* to generate $Q\in{R}^{C^{\prime}\times n\times n}$, $K\in{R}^{C^{\prime}\times n\times n}$ and $V\in{R}^{C\times n\times n}$, respectively, where ${C}^{\prime }$ is smaller than the number of input channels *C* for dimensionality reduction and *n* is the number of 20 common amino acids. Based on $Q$ and $K$, the attention map $A\in{R}^{\left(n+n-1\right)\times \left(n\times n\right)}$ is generated. The affinity operation is as follows:


(8) \begin{equation*} {d}_{i,u}={Q}_u{\varOmega}_{i,u}^T \end{equation*}

where ${d}_{i,u}\in D$ is the degree of correlation between ${Q}_u$ and ${\varOmega}_{i,\kern0.5em u}$, ${Q}_u$ is the feature vector of each feature point in the spatial dimension of $Q$, and ${\varOmega}_u$ is the set of features collected from the same horizontal and vertical directions corresponding to the location of feature point *u* in *K*. Subsequently, the attention feature map $A$ is derived through a SoftMax operation on the channel dimension. The contextual information is collected by an aggregation operation defined as follows:


(9) \begin{equation*} {H}_u^{\prime }=\sum_{i=0}^{\left(n+n-1\right)}{A}_{i,u}{\varPhi}_{i,u}+{H}_u \end{equation*}

where ${H}_u$ represents the feature vector at position u out of the input features. ${A}_{i,u}$ the scalar value at channel *i* and at position *u*. Up to this stage, the feature map ${H}_u^{\prime }$ has acquired a larger context-sensitive field. This enables it to focus on contextual information that is particularly relevant to the current location. This is achieved through a selective focusing on features at other locations that are related to the current amino acid location. This process is made possible by an attention mechanism.

### Implementation

In this study, the PyTorch framework is used to train the model with the Adam optimizer and binary cross-entropy loss function. The hyperparameters, including the epoch number, learning rate, and batch size, are summarized in the Table 3 below. Training the RLEAAI prediction model on an Nvidia A100 GPU with 40 GB of memory takes ~20 hours.

**Table 3:** Hyperparameters used

Hyperparameter	Value
Framework	PyTorch (v2.0.1)
Optimizer	Adam
Loss function	Binary cross-entropy
Epochs	80
Learning rate	0.0005
Batch size	128
Training time	~20 hours
GPU	Nvidia A100 (40GB)

### Evaluation metrics

In order to evaluate the performance of the prediction results, five evaluation metrics are employed in this study: Accuracy (ACC), Precision (Pre), Recall, Specificity (Spe), F1_score (F1), MCC, AUC, and area under the curve for precision recall (AUPR). The equations for these evaluation metrics are provided below:


(10) \begin{equation*} ACC=\frac{TN+ TP}{TN+ TP+ FN+ FP} \end{equation*}

(11) \begin{equation*} Pre=\frac{TP}{TP+ FP} \end{equation*}

(12) \begin{equation*} Recall=\frac{TP}{TP+ FN} \end{equation*}

(13) \begin{equation*} Spe=\frac{TN}{TN+ FP} \end{equation*}

(14) \begin{equation*} F1=\frac{2\ast TP}{2\ast TP+ FN+ FP} \end{equation*}

(15) \begin{equation*} MCC=\frac{2\ast TP}{\sqrt{\left( TP+ FP\right)\ast \left( TP+ FN\right)\ast \left( TN+ FP\right)\ast \left( TN+ FN\right)}} \end{equation*}

where TP and FP represent the number of samples correctly and incorrectly predicted as interaction, respectively. Similarly, TN and FN represent the number of samples correctly and incorrectly predicted as non-interaction, respectively. Furthermore, the area under the curve (AUC) and the AUPR are employed as supplementary assessment metrics. The AUC represents the area under the receiver operating characteristic (ROC) curve, while the area under the precision-recall (PR) curve is referred to as AUPR.

## Results and discussion

### Comparisons of ESM2, one-hot, and ProtT5_XL

For evaluating the performance of ESM2 [31], we employ two other feature embedding methods as controls, i.e. one-hot encoding and another pretrained protein language model ProtT5_XL [30]. One-hot encoding is straightforward but lacks the ability to capture the relationships between amino acids and the surrounding sequence context. ProtT5_XL, leveraging an encoder-decoder framework, is particularly effective for generative tasks like protein design and optimization.

**Figure 3:**
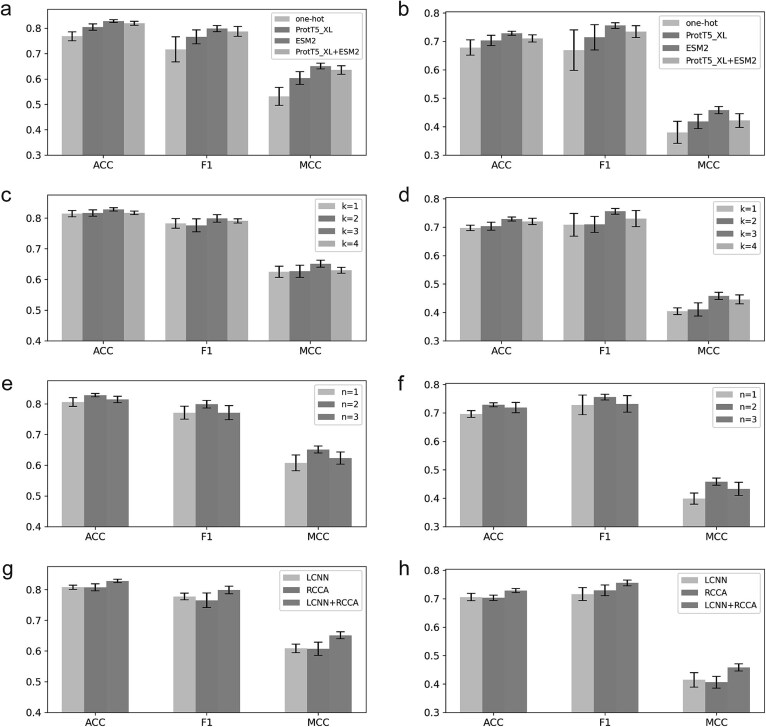
Performance comparison on different parameters**.** (a) Performance comparison of ESM2, one-hot encoding and ProtT5_XL on HIV dataset. (b) Performance comparison of ESM2, one-hot encoding and ProtT5_XL on SARS-CoV-2 dataset. (c) Performance comparison of different *k*-value in CKSAAP on HIV dataset. (d) Performance comparison of different *k*-value in CKSAAP on SARS-CoV-2 dataset. (e) Performance comparison of different LCNN unit number on HIV dataset. (f) Performance comparison of different LCNN unit number on SARS-CoV-2 dataset. (g) Performance comparison of ablation experiments on HIV dataset. (h) Performance comparison of ablation experiments on SARS-CoV-2 dataset.

From Fig. 3a and b ESM2 exhibits a markedly superior performance across all metrics against other two control methods on HIV dataset and SARS-CoV-2 dataset. On the HIV dataset, ESM2 achieves 2.9%, 4.3%, 7.9% improvements in average ACC, F1, and MCC evaluation metrics compared to the pretrained protein language model ProtT5_XL, and 7.8%, 11.5%, 22.5% improvements compared to the one-hot encoding method. On the SARS-CoV-2 dataset, ESM2 achieves 3.6%, 5.8%, 9.5% improvements in average ACC, F1, and MCC evaluation metrics compared to the pretrained protein language model ProtT5_XL, and 7.4%, 12.9%, 20.5% improvements compared to the one-hot encoding method. We also explored the combination of ESM2 and ProtT5 features to enhance model performance. However, the experimental results indicated no significant improvement over using ESM2 features alone. This suggests that ESM2 features might already capture the majority of relevant information for the prediction task, and the addition of ProtT5_XL features may introduce redundant. Notably, as observed in Supplementary Tables S1 and S2, the *P*-values associated with ESM2 are highly significant compared to the other methods, further reinforcing the robustness and reliability of its superiority. These results demonstrate that ESM2 can give more discriminative information than one-hot encoding and ProtT5_XL.

### Determination of parameter *k* value in CKSAAP

In this section, we aim to select a suitable value of parameter *k* in CKSAAP on the HIV dataset. To achieve this, we assess the AAIs prediction performance for various values of *k* (i.e. *k* = 1, 2, 3, 4) on HIV dataset and SARS-CoV-2 dataset. Figure 3c and d illustrates the performance of different *k* values.

As shown in Fig. 3c, on the HIV dataset, when *k* = 3, the values of average ACC, F1, MCC achieve improvements of 1.7%, 2.1%, 4.2% compared to *k* = 1; 1.5%, 2.9%, 3.9% compared to *k* = 2; and 1.4%, 1.1%, 3.3% compared to *k* = 4, respectively. These data demonstrate that *k* = 3 is the suitable choice for CKSAAP in this study. As shown in Fig. 3d, on the SARS-CoV-2 dataset, when *k* = 3, the values of average ACC, F1, MCC achieve improvements of 4.5%, 6.7%, 13.3% compared to *k* = 1; 3.6%, 6.5%, 11.5% compared to *k* = 2; and 1.2%, 3.5%, 2.8% compared to *k* = 4, respectively. As observed in Supplementary Tables S3 and S4, when *k* = 3, the *P*-values are highly significant, reinforcing the effectiveness of this parameter choice for optimizing the model's performance. These data demonstrate that *k* = 3 is the suitable choice for CKSAAP in this study. Therefore, we employ CKSAAP features with *k* = 3 in our subsequent experiments and model design to guarantee that the model can fully leverage the interaction information present in the sequences, thereby enhancing the accuracy and robustness of the prediction.

### Determination of LCNN unit number

To determine the most effective number *n* of LCNN units for prediction, we report the AAIs prediction performance on three different values of *n*, i.e. *n* = 1, 2, and 3.

As shown in Fig. 3e, on the HIV dataset, when *n* = 2, the values of average ACC, F1, MCC achieve improvements of 2.8%, 3.6%, 7.1% compared to *n* = 1, and 1.7%, 3.6%, 4.4% compared to n = 3, respectively. As shown in Fig. 3f, on the SARS-CoV-2 dataset, when n = 2, the values of average ACC, F1, MCC achieve improvements of 4.7%, 3.8%, 14.9% compared to *n* = 1, and 1.4%, 3.3%, 5.9% compared to *n* = 3, respectively. As indicated in Supplementary Tables S5 and S6, when *n* = 2, the p-values are highly significant, further reinforcing the optimal choice of *n* = 2 for maximizing model performance. When *n* = 1, the model has a simpler architecture with fewer parameters, leading to weaker generalization due to its limited learning capacity. When three LCNN units are added (*n* = 3), the model captures deeper features; however, the results show that this increased complexity does not significantly improve performance compared to the model with *n* = 2. This may be attributed to overfitting due to the more complex architecture. These results suggest that two LCNN units configuration effectively balances model complexity and expressive ability, thereby improving predictive performance.

### Performance on combination of LCNN and RCCA

In this study, we employ two modules, i.e. LCNN and RCCA, to fully dig out the discriminative information from the feature representations of antigen and antibody sequences generated by the pretrained protein language model ESM2. To evaluate the importance degree of the modules of LCNN and RCCA, we compare the AAIs prediction performance of LCNN, RCCA, and combination of them (denoted as LCNN+RCCA) on HIV dataset and SARS-CoV-2 dataset. It is noted that, the models of LCNN and RCCA are trained based on the architecture of RLEAAI without RCCA and LCNN, respectively.

As shown in Fig. 3g, on the HIV dataset, the complete model that integrates both the LCNN and RCCA modules achieves the highest performance, with average ACC, F1, and MCC values surpassing the LCNN-only model by 2.6%, 2.7%, and 6.7%, respectively, and the RCCA-only model by 2.5%, 4.4%, and 7.1%, respectively. Similarly, as depicted in Fig. 3h, on the SARS-CoV-2 dataset, the complete model achieves superior results, with average ACC, F1, and MCC values exceeding those of the LCNN-only model by 3.3%, 5.5%, and 10.5%, respectively, and the RCCA-only model by 3.6%, 3.6%, and 12.8%, respectively. As shown in Supplementary Tables S7 and S8, the *P*-values for the combined model are highly significant, further validating the superior performance of the integrated LCNN and RCCA modules over the individual models. Notably, the error bars indicate consistent improvements across multiple runs, confirming the robustness of the complete model.

### Comparison with existing methods on HIV dataset

This section presents a comparative analysis of the AAIs prediction performance of RLEAAI with three state-of-the-art methods, including DeepAAI, S3AI, and AbAgIntPre, on the HIV datasets. The source code for DeepAAI and S3AI can be downloaded from the following https://github.com/enai4bio/DeepAAI and https://github.com/stau-7001/S3AI. To ensure a fair comparison, we have re-trained the prediction models of DeepAAI and S3AI on HIV datasets using the source codes they provided. As AbAgIntPre do not provide source code, the results are generated via feeding the data into its online server (http://www.zzdlab.com/AbAgIntPre) with three different false positive rate (fpr) thresholds. The comparison results of RLEAAI, DeepAAI, S3AI, and AbAgIntPre on three independent datasets are listed in Table 4 and Supplementary Table S1 and S2.

**Table 4:** Performance comparison on HIVtst dataset

Methods	ACC(%)	F1(%)	MCC(%)	AUC(%)	AUPR(%)
DeepAAI	81.31$\pm$0.74	78.30$\pm$1.14	62.09$\pm$1.39	89.57$\pm$0.47	86.89$\pm$0.66
S3AI	78.15$\pm$1.74	74.29$\pm$2.11	55.37$\pm$3.55	85.43$\pm$1.73	82.43$\pm$2.45
AbAgIntPre(fpr = 0.1)	43.66	60.78	–	48.10	42.86
AbAgIntPre(fpr = 0.05)	43.66	60.78	–	48.10	42.86
AbAgIntPre(fpr = 0.01)	43.81	58.91	−2.87	48.10	42.86
RLEAAI	82.94$\pm$0.49	80.26$\pm$1.02	65.36$\pm$0.98	91.01$\pm$0.44	88.94$\pm$0.60

From Table 4 and Supplementary Tables S9 and S10, it is observed that RLEAAI outperforms the three state-of-the-art methods, i.e. DeepAAI, S3AI and AbAgIntPre, concerning all five evaluation indexes, i.e. ACC, F1, MCC, AUC, and AUPR on three independent testing datasets. Concretely, the average ACC, F1, MCC, AUC, and AUPR values of RLEAAI on three datasets are 0.8185, 0.7869, 0.6303, 0.8984, and 0.8727, which are 1.8%, 2.2%, 4.8%, 1.3%, and 1.8% higher than those of DeepAAI and 7.2%, 9.7%, 22.3%, 8.0%, and 10.0% higher than those of S3AI, respectively. These data present that the robustness of RLEAAI is better than three existing state-or-the-art AAI prediction methods.

As an extensive analysis, we perform experiments on label-shuffled data. The results, presented in Supplementary Table S11, reveal that RLEAAI fails to perform effectively without access to true labels, indirectly confirming that the model does not rely on random predictions but instead learns meaningful and valuable knowledge.

### Comparison with existing methods on SARS-CoV-2 dataset

**Figure 4:**
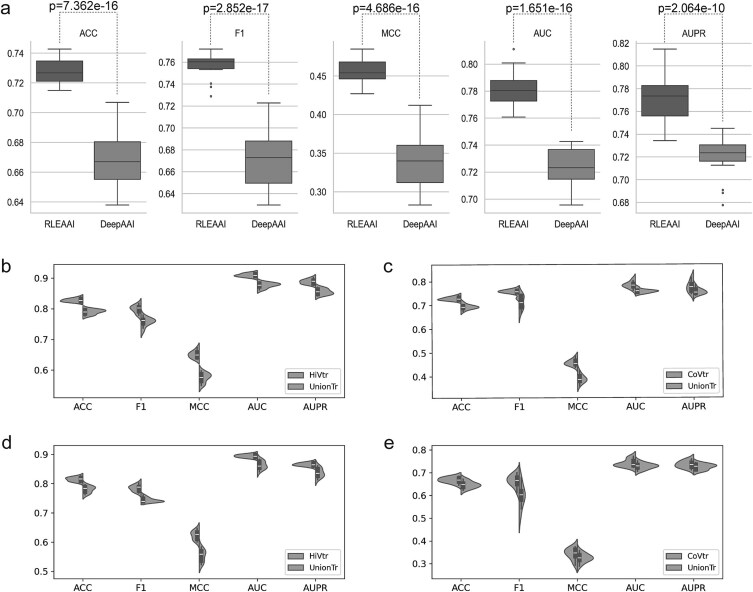
Comparison between RLEAAI and DeepAAI. (a) Box plots of performance between RLEAAI and DeepAAI on the SARS-CoV-2 dataset. (b) HIVtst result of RLEAAI on HIVtr and UnionTr. (c) CoVtst result of RLEAAI on CoVtr and UnionTr. (d) HIVtst result of DeepAAI on HIVtr and UnionTr. (e) CoVtst result of DeepAAI on CoVtr and UnionTr.

To further verify the performance of RLEAAI, we have also compared it with the state-of-the-art AAIs prediction method DeepAAI on the SARS-CoV-2 dataset. On the same training dataset, we separately train 20 different prediction models for RLEAAI and DeepAAI using 20 different initial neural network model parameters. The average performance of RLEAAI and DeepAAI are shown in Fig. 4a.

**Table 5:** Performance comparison on SAbDab dataset

Method	Precision	Recall	Specificity	F1	AUC	AUPR
AbAgIPA	0.810	0.555	0.865	0.654	0.721	0.781
AbAgIntPre	0.666	0.619	0.686	0.640	0.694	0.739
S3AI	0.853	0.863	0.849	0.857	0.938	0.940
DeepAAI	0.842	0.903	0.830	0.872	0.945	0.946
RLEAAI	0.887	0.911	0.884	0.899	0.948	0.939

From Fig. 4a, it is easily found that RLEAAI consistently outperforms DeepAAI concerning all five evaluation indexes, i.e. ACC, F1, MCC, AUC, and AUPR, on the SARS-CoV-2 dataset. Concretely, the ACC, F1, MCC, AUC, and AUPR values of RLEAAI is 0.7280, 0.7573, 0.4568, 0.7813 and 0.7722, which is 9.0%, 12.9%, 35.0%, 7.9%, and 7.2% higher than those of DeepAAI. The p-values calculated by Student’s *t*-test between RLEAAI and DeepAAI are all <10^−10^ on the evaluation indexes of ACC, F1, MCC, AUC, and AUPR, representing that RLEAAI is significantly better than DeepAAI in statistical.

### Performance on model trained on union dataset of HIV and SARS-CoV-2

To evaluate whether the AAIs prediction performance can be improved via combining the training datasets of HIV and SARS-CoV-2 to train the prediction model, we employ RLEAAI and DeepAAI as base methods. Figure 4b–e illustrates the performance comparison between the union training dataset (named UnionTr) and the individual datasets, HIVtr and CoVtr. From Supplementary Table S12 in Supplementary, we observe that the performance of both RLEAAI and DeepAAI on HIVtst and CoVtst is slightly decreased when trained on UnionTr compared to HIVtr and CoVtr. For instance, taking HIVtst as an example, the average ACC, F1, MCC, AUC, and AUPR values of RLEAAI trained on HIVtr are 4.8%, 5.6%, 13.2%, 3.8%, and 3.9% higher, respectively, than those trained on UnionTr. Similarly, DeepAAI shows similar trends.

The performance decrease, particularly in MCC, highlights the challenges of combining datasets from different virus types. Each dataset may contain unique discriminative features specific to HIV or SARS-CoV-2, which can be diluted or conflicting during mixed training. MCC, as a metric sensitive to data imbalance and prediction consistency, is more affected by these challenges. For instance, in HIVtst, the MCC values of RLEAAI and DeepAAI drop significantly when trained on UnionTr, suggesting that the mixed training process struggles to generalize across distinct virus-specific characteristics.

These results suggest that data from different virus types may contain distinct and incompatible discriminative information, and mixed training on UnionTr can potentially weaken the ability of the model to capture these features. Future work could address this limitation by exploring feature separation strategies, weighted training methods, or multi-task learning frameworks that retain virus-specific characteristics while leveraging the combined dataset's size. Additionally, the results underscore the inherent challenges in predicting AAIs. The variability and complexity of AAIs, along with the differences between datasets, pose significant obstacles to achieving generalized and robust performance across diverse viral types.

### Performance comparison on SAbDab dataset

The proposed RLEAAI was compared with the AbAgIPA and AbAgIntPre methods on the SAbDab dataset, and the results are presented in Table 5. The performance of RLEAAI represents the average test results from five-fold cross-validation, while the results of AbAgIPA and AbAgIntPre are derived from the original paper of AbAgIPA. As shown in the table, RLEAAI outperforms AbAgIPA by 9.5%, 64.1%, 2.2%, 38.5%, 31.5%, and 20.2% in terms of Precision, Recall, Specificity, F1 score, AUC, and AUPR, respectively. Similarly, RLEAAI achieves improvements of 33.2%, 47.2%, 28.9%, 40.5%, 36.6%, and 27.1% over AbAgIntPre on the same metrics. Compared to S3AI, RLEAAI demonstrates improvements of ~4.0% in precision, 5.6% in recall, 4.1% in specificity, 4.9% in F1 score, and 1.0% in AUC, while maintaining a similar AUPR. When compared to DeepAAI, RLEAAI shows improvements of around 5.3% in precision, 0.9% in recall, 5.3% in specificity, 3.1% in F1 score, and 0.3% in AUC, again achieving a comparable AUPR.

These significant improvements highlight the effectiveness of RLEAAI in capturing critical interaction features between antibodies and antigens. Particularly, the substantial gains in Recall and F1 score suggest that RLEAAI has a stronger ability to identify true positive interactions while maintaining a balance between precision and recall. This could be attributed to the advanced design of RLEAAI, which leverages enhanced sequence encoding and robust attention mechanisms, enabling it to better generalize across complex datasets like SAbDab.

**Figure 5:**
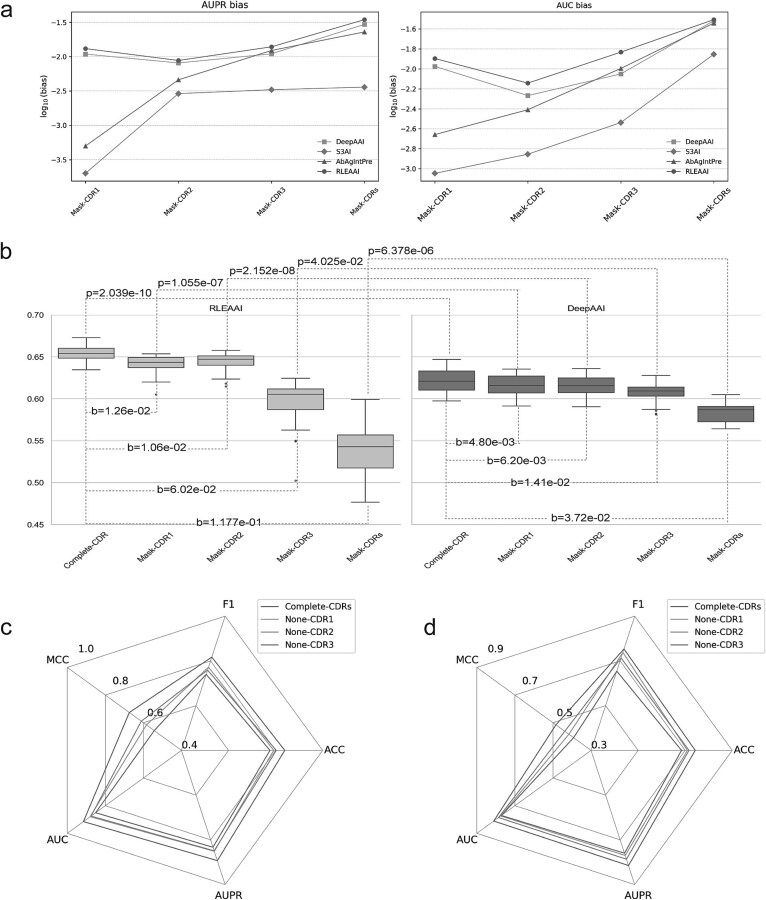
Results on CDR regions. (a) Bias comparison of AUC and AUPR bias on the SARS-CoV-2 test set. (b) Bias comparison of MCC on HIV independent testing set and mask-CDRs corresponds to mask all the CDRs. (c) Performance comparison of models trained with the removal of CDR1, CDR2, or CDR3 in the amino acid sequence on the HIV dataset. (d) Performance comparison of models trained with the removal of CDR1, CDR2, or CDR3 in the amino acid sequence on the SARS-CoV-2 dataset.

### Performance comparison in sensitivity to CDRs

Most of the interactions of antibodies and antigens are based on the specific binding through their variable regions (VRs). Of these, the complementary determining regions (CDRs) are of particular importance in determining the specificity. It has been demonstrated that CDR3 typically plays the most pivotal role in AAIs, while CDR1 and CDR2 contribute to the enhancement of binding specificity and stability. Consequently, evaluating the performance when different CDRs are masked can elucidate the sensitivity of the AAI’s prediction methods to CDRs. Higher sensitivity indicates that the model can discern the essential characteristics of AAIs, thereby enhancing the accuracy of the predictions. In this study, the masking procedure was carried out by occluding specific regions of the amino acid sequence. The condition of no CDRs masked is employed as baseline and the performance bias (abbreviated as *b*) values between baseline and conditions of Mask-CDR1 (only CDR1 is masked), Mask-CDR2 (only CDR2 is masked), Mask-CDR3 (only CDR3 is masked) and Mask-CDRs (all three CDRs are masked) are employed as the sensitivity degrees. The larger bias value, the higher sensitivity. Figure 5 demonstrates the bias values of RLEAAI, DeepAAI, S3AI, and AbAgIntPre on both HIV and SARS-CoV-2 datasets.

From Fig. 5a, it is easily found that the bias values of Mask-CDR1, Mask-CDR2, and Mask-CDR3 are gradually increasing, indicating that CDR1, CDR2, and CDR3 are of increasing importance. RLEAAI achieves the largest bias values of two main evaluation indexes (i.e. AUC and AUPR) among the comparison methods on all conditions (i.e. Mask-CDR1, Mask-CDR2, Mask-CDR3, and Mask-CDRs) for the independent testing dataset of SARS-CoV-2. Concretely, on SARS-CoV-2 dataset, the bias values of Mask-CDR1, Mask-CDR2, Mask-CDR3, and Mask-CDRs concerning the AUC evaluation index of RLEAAI are 0.0127, 0.0072, 0.0147, and 0.0311, which are 19.8%, 33.3%, 65.2%, and 2.6% higher than those of DeepAAI, 1311.1%, 414.3%, 406.9%, and 122.1% higher than those of S3AI and 477.3%, 84.6%, 45.5%, and 8.4% higher than those of AbAgIntPre. Furthermore, Fig. 5b demonstrates the detailed comparison in box plot format between the 20 different prediction models of RLEAAI and DeepAAI on the HIV independent testing dataset concerning the MCC evaluation index. The MCC bias values of Mask-CDR1, Mask-CDR2, Mask-CDR3, and Mask-CDRs of RLEAAI are 162.5%, 70.9%, 326.9%, and 216.4% higher than those of DeepAAI. The similar conclusion results can also be found in Supplementary Figs. S1 and S2. These results demonstrate that RLEAAI is more sensitive to CDR regions than other existing state-or-the-art methods.

To better understand the impact of CDR regions on model performance, we conducted additional experiments by training models with amino acid sequences where CDR1, CDR2, or CDR3 were individually removed. The predictive performance of these models was then compared to that of models trained with the complete CDR sequences. The results, shown in Figs 5c and d, reveal that on the HIV dataset, the MCC values decreased by 15.4%, 10.4%, and 24.2% for models trained with CDR1, CDR2, and CDR3 removed, respectively, compared to the model trained with the complete CDR. On the SARS-CoV-2 dataset, the MCC values decreased by 16.3%, 10.6%, and 23.7% for models with CDR1, CDR2, and CDR3 removed, respectively, when compared to the model trained with the full CDRs.

These results indicate that the presence of CDRs is crucial for the prediction performance. Among the three CDR regions, CDR3 appear to have a more significant impact on the prediction accuracy, as their removal leads to a larger drop in performance. This suggests that these regions may carry more discriminative information for the AAI prediction task. The results also highlight the importance of preserving the full CDRs in order to maintain the predictive power.

## Conclusion

In this study, we introduce a novel deep learning method, RLEAAI, for improving the AAIs’ prediction performance. RLEAAI relies solely on sequence-based representations, which offer the advantage of being broadly applicable to a wide range of antibody–antigen pairs, including those without the structural information resolved through the wet-lab experiments. Despite the progress achieved in the field of protein structure prediction [43, 44], there remains considerable scope for enhancing the accuracy of predicting the structures of antibody and antigen complexes, particularly in the crucial CDR regions [43, 45]. These are the main reasons why RLEAAI is designed based on sequence information only. Based on the pretrained protein language model ESM2, RLEAAI employs two main neural network modules, i.e. LCNN and RCCA, to dig out more discriminative information to learn the final prediction model. In RCCA, the CKSAAP is employed to directly extract the sequence order information. Benchmarked results indicate that, compared to other existing AAI prediction methods, RLEAAI achieves the best performance on both HIV and SARS-CoV-2 datasets with high sensitivities against CDRs.

In future work, we aim to further improve the prediction performance of RLEAAI for AAIs. When a sufficient repository of antibody–antigen complex structures resolved through wet-lab experiments or high-performance structure prediction methods has been amassed, we will incorporate structural information to further enhance prediction accuracy. Additionally, the RLEAAI method will be extended to accommodate a broader range of virus types, e.g. Ebola. We will specifically train the AAI prediction model on the specific antibody types, e.g. IgG and IgM. Although there is still room for optimization, RLEAAI already stands as one of the most accurate tools for AAI prediction, leveraging an advanced protein language model and state-of-the-art deep learning techniques.

## Supplementary Material

suppl_bbaf238(1)

## Data Availability

The data and code are freely accessible at https://github.com/zhouyu9931/RLEAAI.git.
