# Supplementary material for: RLEAAI: improving antibody–antigen interaction prediction using protein language model and sequence order information
Source: Brief Bioinform. 2025 Jun 4;26(3):bbaf238. doi: 10.1093/bib/bbaf238 (PMC12133683; doi:10.1093/bib/bbaf238)
Supplement: suppl_bbaf238(1) [file suppl_bbaf238(1).docx]

RLEAAI: Improving Antibody-Antigen Interaction Prediction Using Protein Language Model and Sequence Order Information

Jun Hu ^1,2, *^, Yu Zhou ^1^, Wen-Yi Zhang ^3, *^, Xiao-Gen Zhou ^1, *^

^1^ College of Information Engineering, Zhejiang University of Technology, Hangzhou, 310023, China.

^2^ Center for AI and Computational Biology, Suzhou Institute of Systems Medicine, Suzhou, 215123, China.

^3^ Westlake AI Therapeutics Lab, Westlake Laboratory of Life Sciences and Biomedicine, 18 Shilongshan Road, Hangzhou, Zhejiang 310024, China.

**Supplementary Information**

**Supporting Texts**

**Text S1.** Composition of K-spaced Amino Acid Pairs (CKSAAP).

CKSAAP^1^ captures the sequence order information by calculating the occurrence frequency of amino acid pairs at different gaps. Given a protein sequence $S=(S_{1}, S_{2}, \ldots,S_{N})$ and corresponding embedding information with dimension $R^{N\times D}$ under a parameter 𝑘 (the maximum gap between amino acid pairs), the feature ($E_{j}(t)$) of the *j-th* pair of amino acid types ${DP}_{j}=(a_{j}^{1},a_{j}^{2})$, $j\in[1,400]$, with gap k is calculated by CKSAAP as:

$E_{j}(t)=\frac{\sum_{i=1}^{N-t-1} \delta_{i}^{a_{j}^{1}}\cdot\delta_{i+t+1}^{a_{j}^{2}}\cdot\frac{emb\left( i \right)+emb\left( i+t+ 1 \right)}{2}}{N-t-1}$ (S1)

where emb(𝑖) is the embedding of the amino acid at position 𝑖, *N* is the length of the sequence *S* and $\delta_{i}^{a_{j}^{1}}$ checks whether the position *i* in the sequence is amino acid $a_{j}^{1}$:

$\delta_{i}^{a_{j}^{1}}=\left\{ \begin{aligned} 1, ifS_{i}=a_{j}^{1} \\ 0, ifS_{i}\neq a_{j}^{1} \end{aligned} \right.$ (S2)

Therefore, the embedding generated by CKSAAP is reshaped into a tensor of with dimension $R^{(K\cdot D+D)\times20\times20}$, which represents the amino acids pair distributions across all positions and gaps.

**Supporting Tables and Figures**

**Table S1.** MCC P-value of ESM2, one-hot encoding and ProtT5_XL on HIV data set.

| - | one-hot | ProtT5_XL | ESM2 | ProtT5_XL+ESM2 |
| --- | --- | --- | --- | --- |
| one-hot | - | 2.86E-05 | 1.57E-07 | 3.36E-07 |
| ProtT5_XL | 2.86E-05 | - | 5.32E-05 | 2.48E-03 |
| ESM2 | 1.57E-07 | 5.32E-05 | - | 2.15E-02 |
| ProtT5_XL+ESM2 | 3.36E-07 | 2.48E-03 | 2.15E-02 | - |

**Table S2.** MCC P-value of ESM2, one-hot encoding and ProtT5_XL on SARS-CoV-2 data set.

| - | one-hot | ProtT5_XL | ESM2 | ProtT5_XL+ESM2 |
| --- | --- | --- | --- | --- |
| one-hot | - | 1.42E-02 | 3.81E-05 | 8.15E-03 |
| ProtT5_XL | 1.42E-02 | - | 3.09E-04 | 7.52E-01 |
| ESM2 | 3.81E-05 | 3.09E-04 | - | 5.00E-04 |
| ProtT5_XL+ESM2 | 8.15E-03 | 7.52E-01 | 5.00E-04 | - |

**Table S3.** MCC P-value of different k-value in CKSAAP on HIV data set.

| - | k=1 | k=2 | k=3 | k=4 |
| --- | --- | --- | --- | --- |
| k=1 | - | 8.29E-01 | 8.26E-04 | 4.22E-01 |
| k=2 | 8.29E-01 | - | 2.90E-03 | 6.28E-01 |
| k=3 | 8.26E-04 | 2.90E-03 | - | 1.49E-04 |
| k=4 | 4.22E-01 | 6.28E-01 | 1.49E-04 | - |

**Table S4.** MCC P-value of different k-value in CKSAAP on SARS-CoV-2 data set.

| - | k=1 | k=2 | k=3 | k=4 |
| --- | --- | --- | --- | --- |
| k=1 | - | 4.36E-01 | 2.67E-09 | 1.64E-06 |
| k=2 | 4.36E-01 | - | 2.46E-05 | 6.70E-04 |
| k=3 | 2.67E-09 | 2.46E-05 | - | 5.89E-02 |
| k=4 | 1.64E-06 | 6.70E-04 | 5.89E-02 | - |

**Table S5.** MCC P-value of different LCNN unit number on HIV data set.

| - | n=1 | n=2 | n=3 |
| --- | --- | --- | --- |
| n=1 | - | 1.68E-04 | 1.31E-01 |
| n=2 | 1.68E-04 | - | 9.76E-04 |
| n=3 | 1.31E-01 | 9.76E-04 | - |

**Table S6.** MCC P-value of different LCNN unit number on SARS-CoV-2 data set.

| - | n=1 | n=2 | n=3 |
| --- | --- | --- | --- |
| n=1 | - | 2.01E-07 | 1.48E-03 |
| n=2 | 2.01E-07 | - | 5.89E-03 |
| n=3 | 1.48E-03 | 5.89E-03 | - |

**Table S7.** MCC P-value of ablation experiments on HIV data set.

| - | LCNN | RCCA | LCNN+RCCA |
| --- | --- | --- | --- |
| LCNN | - | 8.90E-01 | 2.12E-07 |
| RCCA | 8.90E-01 | - | 2.66E-05 |
| LCNN+RCCA | 2.12E-07 | 2.66E-05 | - |

**Table S8.** MCC P-value of ablation experiments on SARS-CoV-2 data set.

| - | LCNN | RCCA | LCNN+RCCA |
| --- | --- | --- | --- |
| LCNN | - | 3.95E-01 | 1.59E-04 |
| RCCA | 3.95E-01 | - | 1.91E-06 |
| LCNN+RCCA | 1.59E-04 | 1.91E-06 | - |

**Table S9**. Performance comparison of RLEAAI, DeepAAI, S3AI and AbAgIntPre on HIVtst90 data set.

| Methods | ACC(%) | F1(%) | MCC(%) | AUC(%) | AUPR(%) |
| --- | --- | --- | --- | --- | --- |
| DeepAAI | 80.46$\pm$0.68 | 76.62$\pm$1.22 | 60.07$\pm$1.28 | 88.74$\pm$0.53 | 85.40$\pm$0.76 |
| S3AI | 76.01$\pm$0.40 | 70.73$\pm$0.41 | 50.52$\pm$0.81 | 82.68$\pm$0.41 | 78.32$\pm$0.45 |
| AbAgIntPre(fpr=0.1) | 42.31 | 59.46 | - | 48.65 | 42.67 |
| AbAgIntPre(fpr=0.05) | 42.31 | 59.46 | - | 48.65 | 42.67 |
| AbAgIntPre(fpr=0.01) | 42.59 | 57.73 | -2.96 | 48.65 | 42.67 |
| RLEAAI | 81.83$\pm$0.56 | 78.26$\pm$1.26 | 62.80$\pm$1.2 | 89.85$\pm$0.57 | 86.74$\pm$0.84 |

**Table S10**. Performance comparison of RLEAAI, DeepAAI, S3AI and AbAgIntPre on HIVtst85 data set.

| Methods | ACC(%) | F1(%) | MCC(%) | AUC(%) | AUPR(%) |
| --- | --- | --- | --- | --- | --- |
| DeepAAI | 79.44$\pm$0.73 | 76.11$\pm$1.30 | 58.33$\pm$1.35 | 87.68$\pm$0.59 | 84.88$\pm$0.81 |
| S3AI | 74.89$\pm$0.41 | 70.13$\pm$0.42 | 48.66$\pm$0.81 | 81.56$\pm$0.42 | 77.52$\pm$0.46 |
| AbAgIntPre(fpr=0.1) | 43.74 | 60.86 | - | 50.83 | 45.62 |
| AbAgIntPre(fpr=0.05) | 43.74 | 60.86 | - | 50.83 | 45.62 |
| AbAgIntPre(fpr=0.01) | 44.62 | 59.62 | 0.23 | 50.83 | 45.62 |
| RLEAAI | 80.79$\pm$0.73 | 77.55$\pm$1.5 | 60.94$\pm$1.51 | 88.67$\pm$0.67 | 85.99$\pm$0.87 |

**Table S11**. The label-shuffled and sequence-shuffled results of RLEAAI on HIV data set.

| Methods | ACC(%) | F1(%) | MCC(%) | AUC(%) | AUPR(%) |
| --- | --- | --- | --- | --- | --- |
| RLEAAI | 82.94$\pm$0.49 | 80.26$\pm$1.02 | 65.36$\pm$0.98 | 91.01$\pm$0.44 | 88.94$\pm$0.60 |
| label-shuffled | 49.71$\pm$0.64 | 45.97$\pm$1.78 | -4.9$\pm$1.25 | 49.94$\pm$0.80 | 50.50$\pm$0.84 |

**Table S12.** The evaluation results for two test sets on mixed-trained models.

| Training set | Testing set | Method | ACC(%) | F1(%) | MCC(%) | AUC(%) | AUPR(%) |
| --- | --- | --- | --- | --- | --- | --- | --- |
| HIVtr | HIVtst | DeepAAI | 81.52$\pm$0.69 | 78.48$\pm$1.03 | 62.37$\pm$1.47 | 89.47$\pm$0.51 | 86.58$\pm$0.55 |
|  |  | RLEAAI | 82.82$\pm$0.55 | 79.88$\pm$1.21 | 65.09$\pm$1.13 | 90.96$\pm$0.54 | 88.87$\pm$0.74 |
| UnionTr | HIVtst | DeepAAI | 78.1$\pm$1.10 | 74.41$\pm$0.76 | 55.33$\pm$2.02 | 86.53$\pm$1.34 | 83.77$\pm$1.49 |
|  |  | RLEAAI | 79.00$\pm$0.80 | 75.67$\pm$1.49 | 57.52$\pm$1.59 | 87.66$\pm$0.84 | 85.55$\pm$0.95 |
| CoVtr | CoVtst | DeepAAI | 66.78$\pm$1.26 | 66.55$\pm$2.45 | 34.22$\pm$2.66 | 74.09$\pm$1.74 | 73.71$\pm$1.49 |
|  |  | RLEAAI | 72.88$\pm$0.70 | 75.59$\pm$1.02 | 45.81$\pm$1.28 | 78.38$\pm$1.37 | 77.57$\pm$2.30 |
| UnionTr | CoVtst | DeepAAI | 64.71$\pm$1.46 | 60.01$\pm$5.03 | 32.56$\pm$1.87 | 72.88$\pm$1.28 | 72.62$\pm$1.23 |
|  |  | RLEAAI | 69.34$\pm$0.97 | 71.25$\pm$2.76 | 38.96$\pm$1.77 | 75.75$\pm$0.76 | 75.62$\pm$1.24 |


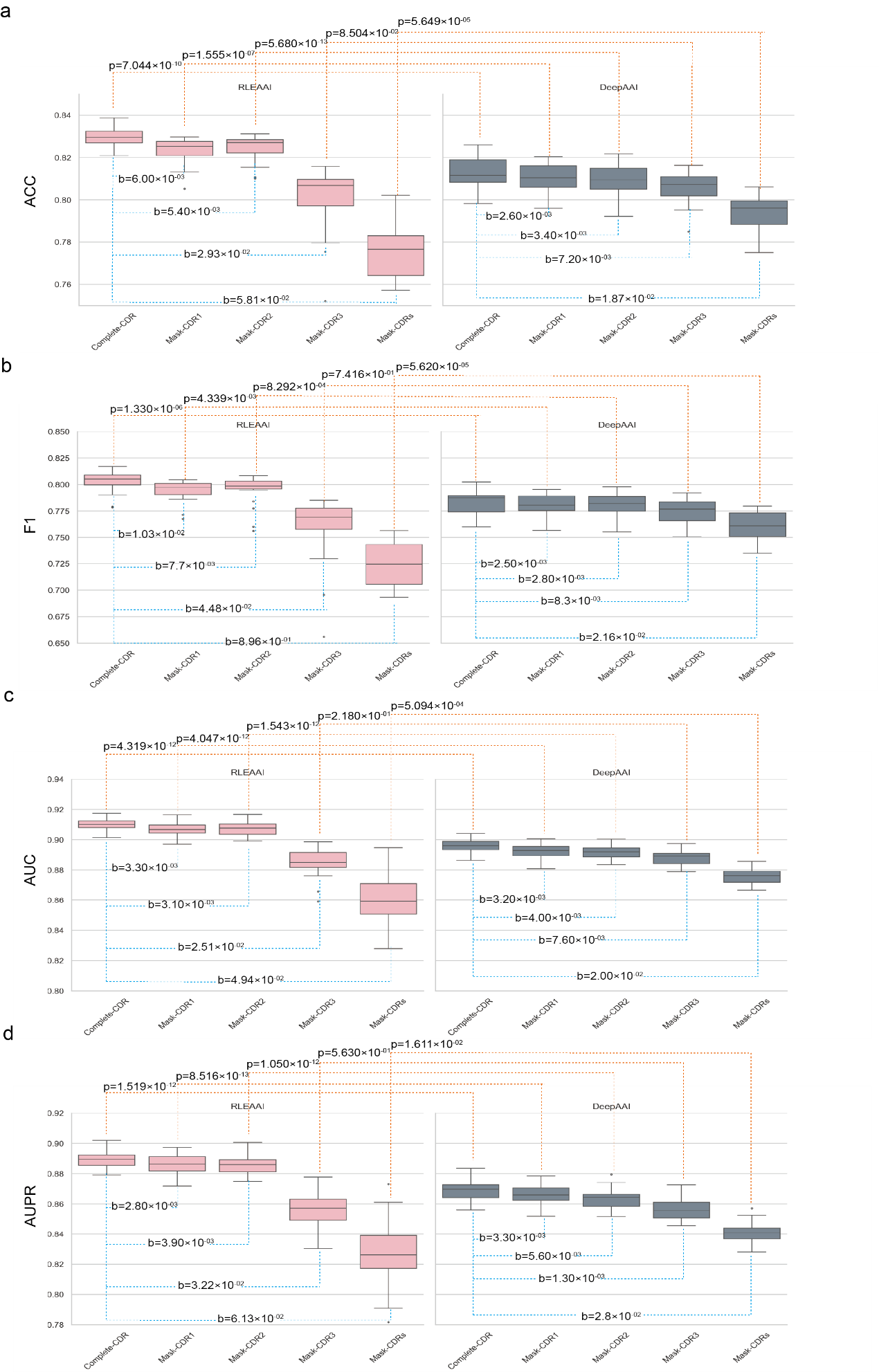


**Figure S1.** Bias comparisons of RLEAAI and DeepAAI when different CDRs are masked on different evaluation indexes. (**a**) on ACC. (**b**) on F1. (**c**) on AUC. (d) on AUPR.


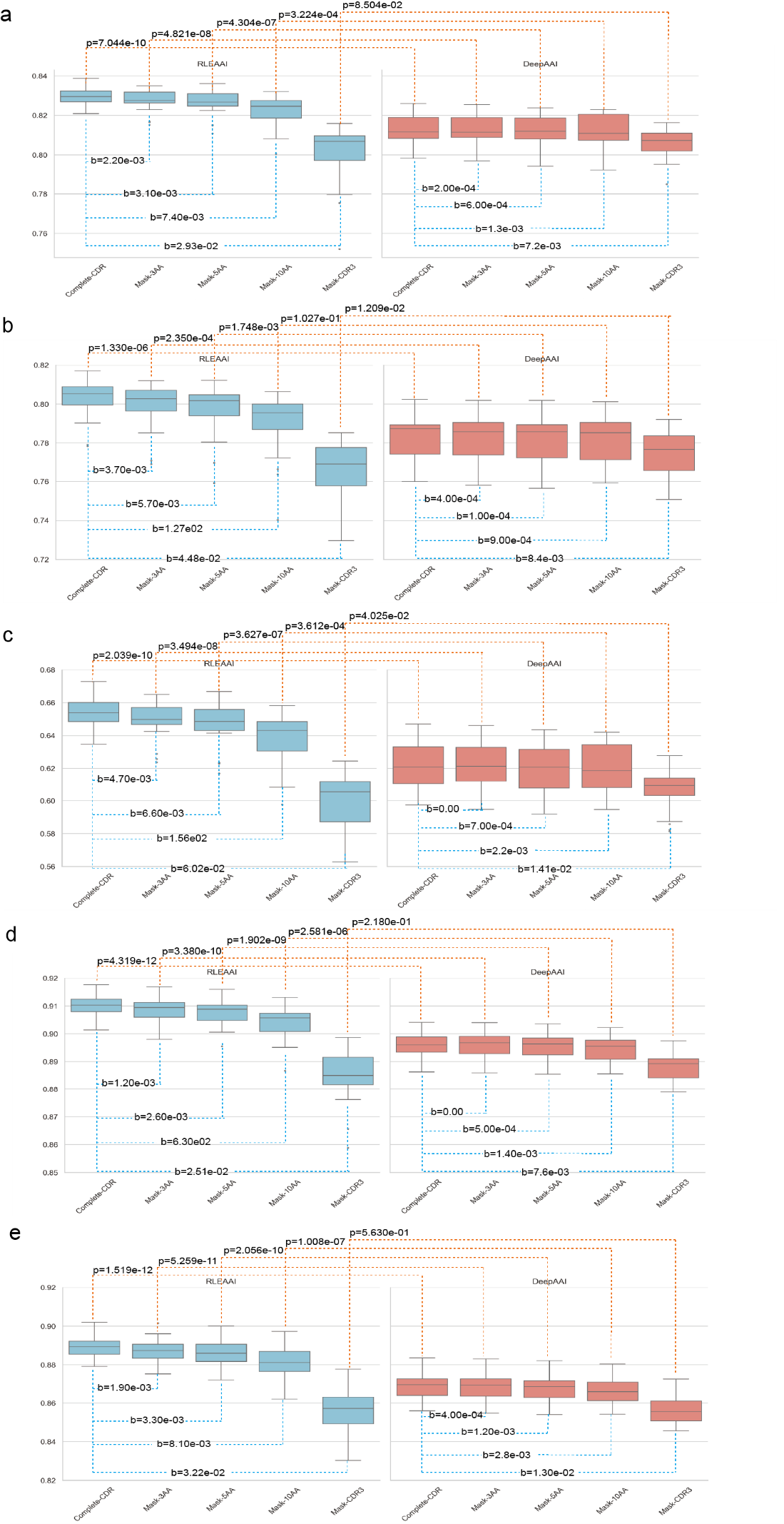


**Figure S2.** Bias comparisons of RLEAAI and DeepAAI when different numbers amino acids in CDR3 are replaced by the unknown amino acid X on different evaluation indexes. (**a**) on ACC. (**b**) on F1. (**c**) on AUC. (d) on AUC. (e) on AUPR.

**REFERENCES**

(1) Chen, Z.; Zhou, Y.; Song, J.; Zhang, Z. hCKSAAP_UbSite: improved prediction of human ubiquitination sites by exploiting amino acid pattern and properties. *Biochimica et Biophysica Acta (BBA)-Proteins and Proteomics* **2013**, *1834* (8), 1461-1467.
